# Opposing Roles of CREPT and p15RS in Tumorigenesis via Differential Regulation of Wnt Signaling

**DOI:** 10.3390/cancers18121911

**Published:** 2026-06-11

**Authors:** Dekang Zhou, Jun Li, Fangli Ren, Yajun Cao, Bobin Ning, Wenchen Wang, Baoqing Jia, Guo-Min Li, Yinyin Wang, Zhijie Chang

**Affiliations:** 1 State Key Laboratory of Membrane Biology, School of Basic Medical Sciences, School of Medicine, Tsinghua University, Beijing 100084, China; 2 Tsinghua-Peking Joint Center for Life Sciences, School of Life Science, Tsinghua University, Beijing 100084, China; 3 Department of General Surgery, The First Medical Center, PLA General Hospital, Beijing 100853, China; 4 Chinese Institute for Cancer Research, Chinese Institutes for Medical Research, Beijing 100069, China; 5 School of Basic Medical Sciences, Capital Medical University, Beijing 100069, China

**Keywords:** CREPT/RPRD1B, p15RS/RPRD1A, tumorigenesis, Wnt signaling, protein oligomerization

## Abstract

**Background/Objectives:** Tumors are induced by overactivation of oncogenes and loss of tumor suppressor genes. Recently, a family of proteins containing CID (C-terminal domain (CTD)-interacting domain) domains, named CREPT/RPRD1B and p15RS/RPRD1A, has been identified to be involved in tumorigenesis through the regulation of the cell cycle. Interestingly, while p15RS was shown to inhibit cell proliferation, CREPT was demonstrated to promote tumorigenesis by accelerating tumor cell cycle progression. **Methods:** To decipher why these two proteins function oppositely, we aimed to reveal the disparities in their clinical outcomes and protein properties. **Results:** We observed that CREPT and p15RS are both highly expressed in tumors, but with opposite prognostic implications. We confirmed that CREPT promotes, but p15RS inhibits cell proliferation via regulation of Wnt/β-catenin signaling activation. The CID domain of CREPT differs from that of p15RS in conformation and charge distribution. CREPT exhibits a significantly stronger oligomerization capacity than p15RS, which is mediated by the CCT (coiled-coil terminus) domain. We demonstrated that the differences in both CID and CCT domains between CREPT and p15RS contribute to their opposite physiological functions. **Conclusions:** In conclusion, our results demonstrate that despite high primary sequence similarity, CREPT and p15RS exhibit distinctive biochemical properties. These differences ultimately explain their functional divergence in tumorigenesis and offer novel insights into CREPT-targeted drug design.

## Simple Summary

CREPT and p15RS share high sequence similarity, but play divergent roles in tumorigenesis. However, the mechanism underlying their functional divergence remains unclear. In this study, we performed comparative analyses and found that CREPT promotes tumorigenesis, whereas p15RS suppresses it through opposing regulation of Wnt signaling. Specifically, we demonstrated that their conserved CID and CCT domains display distinct biochemical properties, which account for their opposite functions in cancer development.

## 1. Introduction

Tumorigenesis occurs due to aberrant expression of multitudinous genes, which are typically categorized into either overactivated oncogenes or silenced tumor suppressors [1]. In general, oncogenes promote, but tumor suppressor genes inhibit cell proliferation [2]. Although oncogenes and tumor suppressor genes seem to belong to distinctive categories, they can actually be part of the same protein family and exhibit significant similarities in terms of sequence or domain structure. For instance, c-Jun and JunB, two homologue proteins belonging to the AP-1 transcription factor family, exhibit positive and negative effects on cell proliferation and tumorigenesis, respectively [3,4]. In addition, p53, p63, and p73 are all members of the p53 protein family. However, unlike p53, which is typically involved in tumor suppression, p63 and p73 were shown to promote tumorigenesis [5,6]. All these cases indicated that proteins with similar structure might function differently during tumorigenesis.

As a result of dysregulated oncogenes or tumor suppressor genes, aberrant activation of proliferation signaling is one of the hallmarks of cancer [7]. Wnt signaling is frequently overactivated in multiple types of tumors especially in colorectal adenocarcinoma [8]. Under quiescent conditions, cytosolic β-catenin is maintained at a low level. In this context, nuclear TCF/LEF transcriptional factors form a repressive complex with Groucho, resulting in the silence of Wnt target genes [9,10]. In the presence of Wnt ligands, β-catenin accumulates and translocates into the nucleus. It subsequently replaces Groucho to form an active complex with TCF to promote the expression of downstream genes including *CCND1* and *Myc* [11]. Mutation, amplification, or deletion of Wnt cascade components may cause constitutive activation of cell proliferation and dysregulate cell cycle progression, ultimately leading to tumorigenesis [12,13]. For example, inactivating mutations of APC and activating mutations of β-catenin frequently occur in colorectal cancer, hepatocellular carcinoma, and gastric cancer [8]. Therefore, developing therapies targeting Wnt/β-catenin has been widely investigated in various cancers [14].

Our previous studies have identified that CREPT (cell cycle-related and expression-elevated protein in tumor, also named RPRD1B) is a novel oncogene that is highly expressed across multiple types of tumors and correlates with unfavorable prognosis [15,16,17,18]. Overexpression of CREPT has been observed to result in accelerated cell cycle, enhanced cell proliferation and increased clonogenic ability in a variety of tumor cells [19,20,21]. Mechanistically, CREPT facilitates DNA loop formation via interaction with RNA polymerase II (Pol II) and promotes malignant gene transcription [15,22]. In addition, it has also been reported that CREPT enhances Wnt/β-catenin signaling by facilitating the formation of β-catenin-TCF4 transcription activator complex [23,24] and p300 mediated β-catenin stabilization [25].

On the other hand, p15RS (p15-related sequence, also named RPRD1A), the paralogous protein of CREPT in fish, mouse, and human [25], has been demonstrated to act as a tumor suppressor since its first identification in 2002 [26]. Overexpression of p15RS downregulates cyclin D1 expression, leading to cell cycle arrest and suppressed malignancy [26,27,28]. In contrast to CREPT, p15RS exhibits an inhibitory effect on Wnt signaling. It is reported that p15RS disarms β-catenin-TCF4 interaction [29]. In addition, p15RS is able to recruit HDAC2 to maintain the deacetylation status of histone H3 [30], thereby functioning as a negative regulator of Wnt signaling.

Accumulating evidence has demonstrated the opposite role of CREPT and p15RS in tumorigenesis and Wnt/β-catenin signaling. However, some controversial results have been reported by other groups. For example, it has been shown that p15RS is highly expressed in hepatocellular carcinoma and contributes to its progression [31]. Additionally, both CREPT and p15RS interact with Pol II [32,33,34], but their respective contributions to transcription are still controversial [35,36].

Intrigued by the high similarity of these two proteins with distinct functions, we conducted a systematic study to comprehensively compare the functions and mechanisms of CREPT and p15RS in tumorigenesis. We observed that both CREPT and p15RS are highly expressed in tumor tissues. However, CREPT promotes cell proliferation and Wnt signaling, while p15RS exerts an inhibitory role in these processes. Moreover, by constructing domain-swapping proteins, we identified that the CID domains of CREPT and p15RS determine their respective functions due to their distinctive conformation and charge distribution. The CCT domains are responsible for protein oligomerization, but the CPEPT CCT domain exhibits a much stronger propensity for mediating oligomer formations. Altogether, we revealed the similarities and differences of CREPT and p15RS in tumorigenesis.

## 2. Materials and Methods

### 2.1. Plasmid and Reagents

Flag/HA/Myc-CREPT, Flag/HA/Myc-p15RS, Flag-CPP, Flag-PCC, Flag-CCP, Flag-PPC, Flag-CREPT-RPR/CCT, and Flag-p15RS-RPR/CCT expression plasmids were constructed based on the pcDNA3.1 backbone. SuperTop-Flash, pRL-TK, and Wnt1 plasmids were used as previously described [23,30,37]. Site-directed mutations were generated using a Fast Site-Directed Mutagenesis Kit (Tiangen Biotech, Beijing, China). Anti-HA (sc-7392), and anti-β-tubulin (sc-5274) antibodies were purchased from Santa Cruz Biotechnology, Dallas, TX, USA. Anti-c-Myc (9402S) and anti-cyclin D1 (55506S) antibodies were purchased from Cell Signaling Technology, Danvers, MA, USA. Anti-Flag (F3165) antibody was purchased from Sigma, St. Louis, MO, USA. Anti-p15RS (23652-1-AP) antibody was purchased from Proteintech, Wuhan, China. Anti-CREPT antibody was produced in our laboratory. Fluorescent secondary antibodies were purchased from ZSGB-bio, Beijing, China. Wnt3a-conditioned medium (CM) was prepared as previously described [23]. Protein-G agarose beads were purchased from Cytiva, Wilmington, DE, USA. Anti-DYKDDDDK Affinity Beads were purchased from Smart-Lifesciences, Changzhou, China.

### 2.2. Cell Culture and Transfection

HEK-293T, SW480, MGC803, and Huh7 cells were cultured in Dulbecco’s modified Eagle’s medium (DMEM) supplemented with 10% fetal bovine serum (FBS). DLD1 cells were cultured in RPMI-1640 medium with 10% FBS. All cells were maintained at 37 °C in a humidified atmosphere with 5% CO2. All cell lines were obtained from the National Infrastructure of Cell Line Resources, with corresponding accession numbers listed as follows: 293T (1101HUM-PUMC000091), SW480 (1101HUM-PUMC000166), MGC803 (1101HUM-PUMC000660), Huh7 (1101HUM-PUMC000679), and DLD1 (1101HUM-PUMC000671). Plasmids were transfected into cells using VigoFect (Vigorous Biotechnology, China) reagents according to the manufacturer’s instructions.

### 2.3. Cell Proliferation Assay

Cell proliferation was measured using the CCK-8 assay (CK04, DOJINDO Laboratories, Kumamoto, Japan). Briefly, 500–3000 cells were seeded into 96-well plates, with six replications according to the cell type and experimental aims. After incubation for the indicated time, 10% (*v*/*v*) CCK-8 reagent was added to each well and incubated for another 3 h, then the absorbance was measured at 450 nm.

### 2.4. Colony Formation Assay

A total of 1000 cells were seeded in triplicate into 6-well plates and cultured for 12 days. During this period, the medium was refreshed every 3 days. Then, cells were fixed in absolute methanol for 20 min, followed by 0.1% crystal violet staining for 1 h at room temperature. Clonogenic ability was quantified using ImageJ (version 1.54p) software based on colony area.

### 2.5. Serum Starvation and Stimulation

SW480 and MGC803 cells were seeded into 6-well plates at 25% confluence and cultured in complete medium for 24 h until fully adherent. Then, cells were washed 3 times with PBS and starved in serum-free medium for 12 h. After that, cells were stimulated with fresh serum-containing medium for an additional 6 h prior to harvest.

### 2.6. Immunofluorescence (IF) and Immunohistochemistry (IHC)

Clinical tissues were collected from the Chinese PLA General Hospital with ethics approval from its institutional ethics committee. IF and IHC experiments were performed according to the antibody supplier’s protocol. For both assays, primary antibodies were diluted 1:50 for CREPT and 1:100 for p15RS, and all antibody dilutions were optimized by preliminary experiments. The secondary antibody was diluted 1:1000 for IF, while a ready-to-use formulation was applied directly for IHC. IHC slices were scanned by an automatic digital slide scanning system (KFBIO, Ningbo, China). IF images were acquired with a confocal microscope (FV3000, Evident Scientific, Tokyo, Japan) using a 60× oil immersion objective.

### 2.7. Luciferase Assay

In a 24-well plate, SuperTop-Flash, pRL-TK, and the indicated expression plasmid were co-transfected into HEK-293T cells. Additional Wnt1 plasmid was added if the Wnt signaling needed to be activated. Alternatively, Wnt3a conditioned medium was used instead of Wnt1 plasmid transfection to activate Wnt signaling. At 30 h after transfection, cells were harvested and lysed in 1 × ULB buffer. Luciferase activity was measured following the manufacturer’s manual of the Dual-Lucy Assay Kit (Vigorous Biotechnology, Beijing, China) using a Varioskan Flash (Thermo Scientific, Waltham, MA, USA) microplate reader. Firefly luciferase activity was normalized against Renilla luciferase activity to calculate the relative luciferase signal.

### 2.8. Immunoprecipitation (IP) and Western Blotting (WB)

For IP, HEK-293T cell were cultured in 60 mm dishes, and indicated plasmids were co-transfected into the cells. At 30 h after transfection, cells were harvested and lysed in RIPA buffer for 20 min on ice. After centrifugation, the supernatant was transferred into a new tube and incubated with indicated antibody and protein G agarose beads at 4 °C overnight. The beads were washed five times with cell lysis buffer, followed by denaturing or non-denaturing elution depending on the experimental objectives. For Western blotting, cells were lysed in RIPA buffer. Total protein concentration was measured using Bradford reagents (Thermo Scientific, USA). An appropriate amount of protein was separated by SDS-PAGE and transferred to a PVDF membrane. The membrane was incubated with specific antibodies, then washed with TBST prior to luminescence detection.

### 2.9. Prokaryotic Protein Purification and Dynamic Light Scattering (DLS) Assay

Human CREPT and p15RS coding sequences were cloned into the pET-22b plasmid. BL21 (DE3) harboring expression plasmids were incubated at 37 °C in LB medium. When OD_600_ reached 0.8, the culture was induced by addition of 0.5 mM IPTG and further incubated at 16 °C overnight. Cells were harvested and lysed in lysis buffer (50 mM NaH_2_PO_4_, 300 mM NaCl, 10 mM imidazole, pH 8.0). After centrifugation at 12,000× *g* at 4 °C for 1 h, the supernatant was applied to a Ni-NTA affinity chromatography column. The column was washed with washing buffer (50 mM Tris-HCl, 500 mM NaCl, 30 mM imidazole, pH 8.0) and then eluted with elution buffer (50 mM Tris-HCl, 300 mM NaCl, 300 mM imidazole, pH 8.0). The eluted proteins were further purified by a Superdex 200 gel filtration column in running buffer (25 mM Tris-HCl, 150 mM NaCl, pH 8.0). For DLS assays, all proteins were adjusted to a concentration of 2 mg/mL in the gel filtration running buffer. DLS data were collected in NynaPro (Wyatt, Santa Barbara, CA, USA) and averaged for 30 acquisitions.

### 2.10. Cross-Linking Assay

HEK-293T cells were transiently transfected with expression plasmids and harvested after 48 h. Cells were lysed with prechilled TGH buffer (20 mM HEPES, 10% glycerol, 0.1% Triton X-100, pH 7.5) on ice for 20 min, followed by 20 min centrifugation. The supernatant was collected, and formaldehyde with a final concentration of 0.1% or 1% (*v*/*v*) was added. Cell lysates or purified protein were incubated with formaldehyde at room temperature for the indicated time. Then, the reaction was stopped by adding SDS-PAGE loading buffer and boiled for 10 min.

### 2.11. RNA Isolation and Real-Time PCR (RT-PCR)

At least 1 × 10^6^ cells were used for RNA isolation by TRIzol reagent (Thermo Scientific, USA), and 2 μg total RNA was used for reverse transcription. Then, RT-PCR was performed to measure specific gene expression by Talent qPCR PreMix kit (Tiangen Biotech, China) under the following conditions: denaturing, 95 °C, 5 s; annealing, 58 °C, 10 s and extension, 72 °C, 15 s. The primer sequences are provided in Supplemental Table S1.

### 2.12. Software

Phylogenetic analysis was performed using MEGA7 software [38]. The phylogenetic tree, gene structure, and domain structure were visualized by GSDS 2.0 [39]. Structural demonstration and surface electrostatic potential calculation were performed by ChimeraX [40]. TCGA data were downloaded from the UCSC Xena database (https://xena.ucsc.edu/) [41]. Survival analysis was performed based on the UCSC Xena or OncoLnc database (http://www.oncolnc.org/) [42]. All experiments in this study were repeated at least 3 times. All data are expressed as means ± standard error of the mean (SEM). A two-tailed unpaired Student’s *t*-test was used to determine significant differences between the two groups. The results were analyzed and visualized by R (version 4.6.0) or GraphPad Prism (version 10.1.2) software. *** *p* < 0.001, ** *p* < 0.01, * *p* < 0.05, n.s., no significance.

## 3. Results

### 3.1. CREPT and p15RS Are Highly Related in Evolution

**Figure 1:**
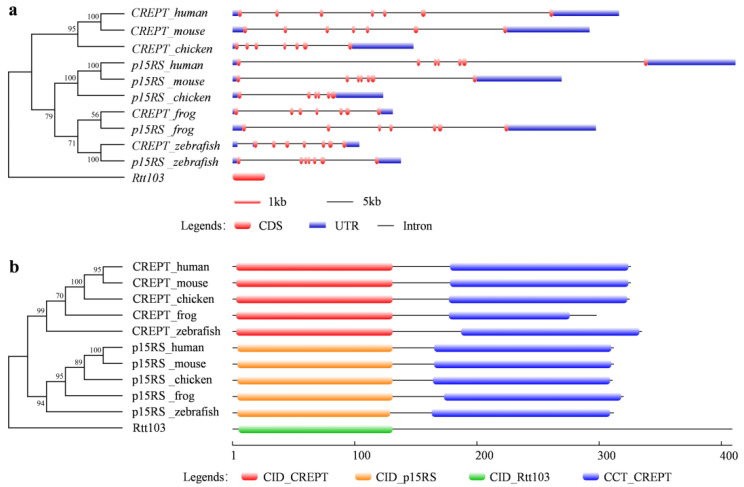
Phylogenetic analysis, gene structure and domain architecture of CREPT and p15RS. (**a**) Phylogenetic analysis and structure illustration of *CREPT* and *p15RS* orthologues across different species. *Rtt103* was selected as the outgroup root. (**b**) Phylogenetic analysis and domain architecture of CREPT and p15RS proteins across different species. Rtt103 was selected as the outgroup root. Multiple sequence alignment was performed using ClustalW in MEGA7. Phylogenetic trees were generated by the neighbor-joining algorithm with 1000 bootstrap replicates.

To study the differences between CREPT and p15RS, we constructed phylogenetic trees based on gene and protein sequences in this family from various species (Figure 1a,b). Gene structure analysis showed that *CREPT* and *p15RS* transcripts from the selected species contain seven exons and six introns, with the exception of chicken *p15RS* and yeast *Rtt103*. The results showed that CREPT and p15RS shared high similarity at both the gene and protein levels. Interestingly, it appeared that CREPT and p15RS evolved from the yeast gene *Rtt103*. All the proteins contain a CID domain, which is highly conserved across species, although the CID domains of CREPT and p15RS showed slight differences. Interestingly, both CREPT and p15RS contained a CCT domain, but Rtt103 lacked this domain.

### 3.2. CREPT and p15RS Are Both Highly Expressed in Colon Cancer

**Figure 2:**
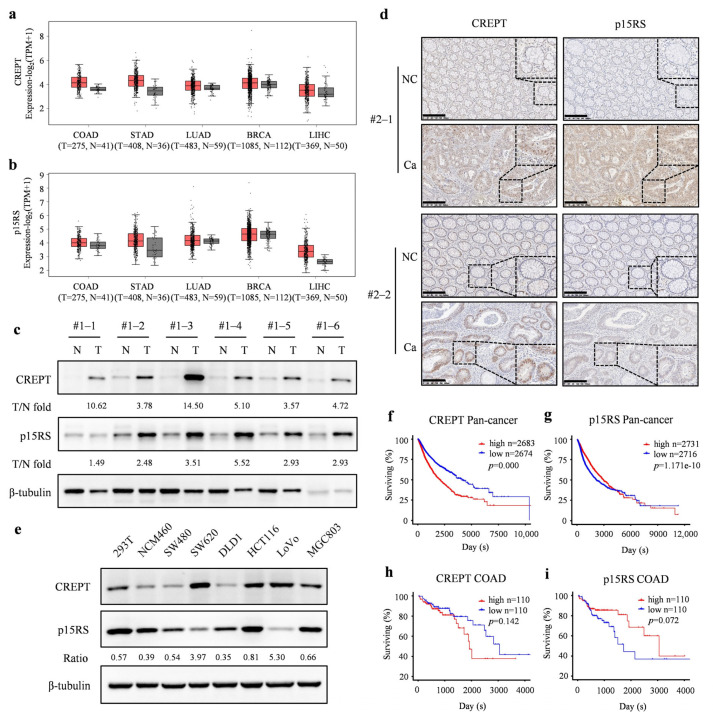
CREPT and p15RS are both overexpressed in colon cancer, but correlate with distinct clinical prognosis. (**a**,**b**) TCGA data showed that CREPT (**a**) and p15RS (**b**) were both upregulated in tumor tissues across multitype cancer types. Red bars indicate tumor tissues, and black bars indicate normal tissues. (**c**) Protein expression levels were evaluated in paired normal tissues and cancer tissues obtained from colon cancer patients. Patient IDs are labeled above the lanes. N represents normal tissues, and T represents tumor tissues. T/N fold: tumor/normal expression fold change normalized to the loading control in paired samples. (**d**) Immunohistochemical staining showed that CREPT and p15RS were both highly expressed in clinical colon tumor tissues. Patient IDs are labeled at the left side. NC: normal tissue control; Ca: cancer tissue. Scale bar, 200 μm. (**e**) Protein expression levels of CREPT and p15RS in the selected cell lines. The CREPT/p15RS expression ratio is labeled in the middle of each lane. (**f**,**g**) Pan-cancer overall survival stratified by the expression level of CREPT (**f**) or p15RS (**g**). Data were obtained from the Xena database. *p*, Log-rank *p*-value. (**h**,**i**) Colon cancer overall survival stratified by the expression level of CREPT (**h**) or p15RS (**i**). Data were obtained from the OncoLnc database. *p*, Log-rank *p*-value.

To decipher the function of CREPT and p15RS during tumorigenesis, we determined to examine their expression levels using the GEPIA2 website (http://gepia2.cancer-pku.cn/) [43] based on TCGA. We examined five representative tumors—colon cancer (COAD), stomach cancer (STAD), lung adenocarcinoma (LUAD), breast cancer (BRCA), and liver cancer (LIHC)—and found that both CREPT and p15RS were upregulated in tumors compared with normal tissues (Figure 2a,b). To validate these findings, we detected the protein levels of CREPT and p15RS in clinical samples obtained from colon cancer patients. The results showed that compared with the paired adjacent normal tissues, both CREPT and p15RS were highly expressed in tumor tissues. However, CREPT exhibited more pronounced upregulation in tumors compared with p15RS (Figure 2c). Similarly, our immunohistochemistry staining results showed that CREPT and p15RS, which mainly localized in the nucleus, were both highly expressed in tumor sections (Figure 2d).

Next, we examined CREPT and p15RS expression levels in a panel of normal and cancer cell lines. HEK-293T and NCM460 are immortalized normal cell lines, while SW480, SW620, DLD1, HCT116, LoVo, and MGC803 are cancer cell lines. The results showed abundant expression of both CREPT and p15RS in both normal and tumor cells, with varying expression patterns among different cell lines (Figure 2e). Intriguingly, we observed that the CREPT/p15RS ratio reached the highest level in SW620 and LoVo, two metastatic tumor cell lines, while the ratio was significantly lower in other non-metastatic or normal cell lines (Figure 2e, see “ratio” in the middle). This finding implies that an increased ratio of CREPT to p15SR may correlate with tumor metastasis.

To investigate the correlation between the expression of CREPT and p15RS and clinical outcomes, we stratified pan-cancer and COAD samples from TCGA according to their expression levels using 25% as the threshold. The results showed that patients with high CREPT expression had shorter overall survival, whereas the patients with high p15RS expression exhibited longer overall survival in both pan-cancer and COAD cohorts (Figure 2f–i). These results demonstrated that CREPT and p15RS oppositely correlated with patient survival, echoing our previous studies showing that CREPT promoted while p15RS inhibited tumor growth [15,37].

### 3.3. CREPT Promotes, but p15RS Inhibits Cell Proliferation and Clonogenicity

**Figure 3:**
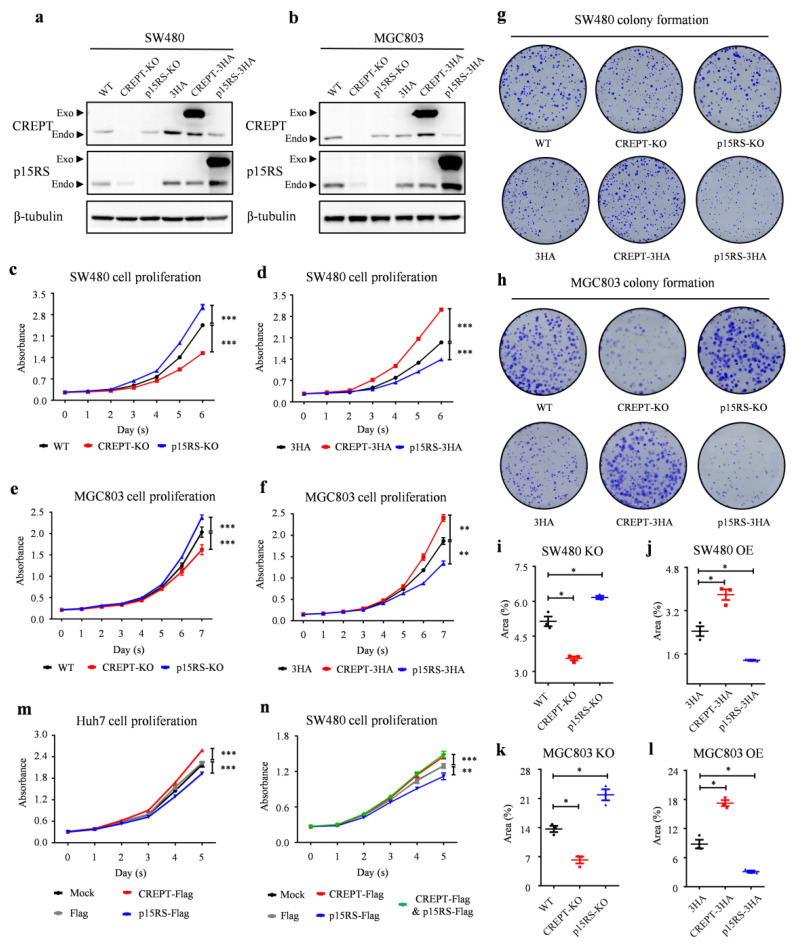
CREPT promotes, but p15RS inhibits cell proliferation and colony formation. (**a**,**b**) Protein levels of CREPT and p15RS in CREPT or p15RS KO or overexpression stable cell lines derived from SW480 (**a**) or MGC803 (**b**) cell lines. (**c**,**d**) Proliferation rates of SW480 cells with CREPT/p15RS KO (**c**) or overexpression (**d**). (**e**,**f**) Proliferation rates of MGC803 cells with CREPT/p15RS KO (**e**) or overexpression (**f**). (**g**,**h**) Representative images of plate colony formation assays for CREPT/p15RS KO or overexpression cells derived from SW480 (**g**) or MGC803 (**h**). (**i**–**l**) Quantification of colony formation assays for SW480 (**i**,**j**) and MGC803 (**k**,**l**) cells. (**m**) Proliferation rates of Huh7 cells after transient transfection with the indicated plasmids. (**n**) Proliferation rates of SW480 cells after transient transfection with the indicated plasmids. *** *p* < 0.001, ** *p* < 0.01, * *p* < 0.05.

Since high expression of CREPT and p15RS correlated with distinct cancer prognosis trends, we speculated that CREPT and p15RS might have distinct roles in cell proliferation and colony formation. To this end, we generated several stable cell lines with knockout (KO) or overexpression (OE) of either CREPT or p15RS using the colon cancer cell line SW480 (Figure 3a) and gastric cancer cell line MGC803 (Figure 3b). We performed CCK-8 assays in parallel to examine cell proliferation rates. The results showed that deletion of CREPT dramatically reduced cell growth rates, while deletion of p15RS accelerated cell growth (Figure 3c). Reciprocally, overexpression of CREPT promoted cell proliferation, whereas overexpression of p15RS repressed cell growth (Figure 3d). Similar results were observed in MGC803 cells (Figure 3e,f). These results suggest that CREPT promotes while p15RS inhibits tumor cell proliferation.

Next, we examined the colony formation ability of the cells. The results showed that deletion of CREPT or overexpression of p15RS impaired the colony formation, while deletion of p15RS or overexpression of CREPT augmented the colony numbers dramatically (Figure 3g,h). A quantitative examination showed these effects to be statistically significant (Figure 3i–l).

We finally verified their opposite functions by transiently overexpressing CREPT or p15RS in Huh7, a hepatocellular carcinoma cell line (Figure 3m). These results were consistent with the role of CREPT and p15RS in stable cell lines regarding cell proliferation control. In particular, when CREPT and p15RS were simultaneously overexpressed at comparable levels in SW480, the double-OE cells exhibited enhanced cell proliferation similar to that of CREPT OE cells (Figure 3n). These results indicated that CREPT played a dominant role in promoting cell proliferation compared with the inhibitory role of p15RS.

Collectively, we conclude that the promotive effects of CREPT and the inhibitory effects of p15RS on cell growth are conserved across various cancer types. The overall functional outcome may be determined by their relative expression ratios.

### 3.4. CREPT Enhances, but p15RS Inhibits Wnt/β-Catenin Signaling

**Figure 4:**
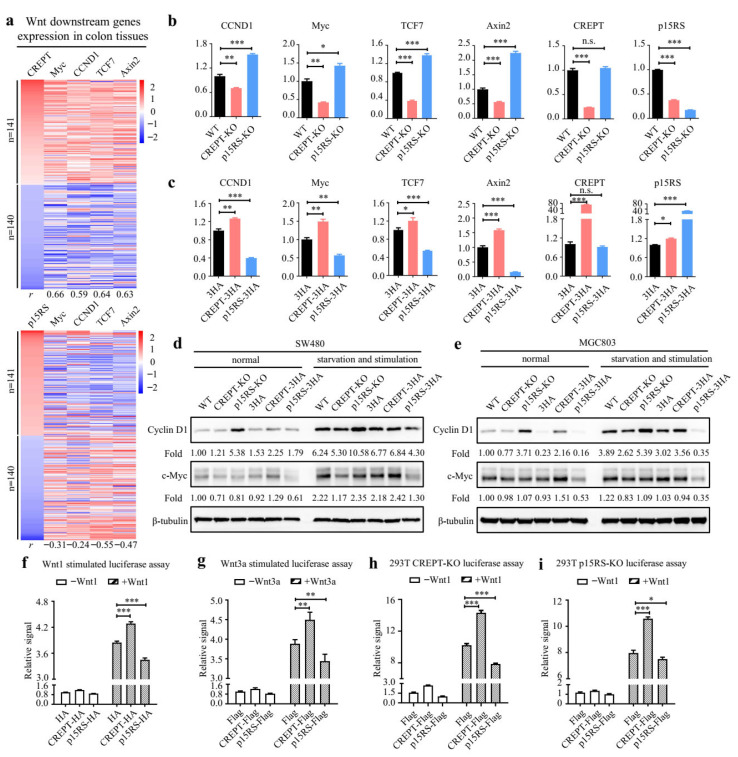
CREPT promotes, but p15RS inhibits Wnt/β-catenin signaling. (**a**) Correlation analysis of CREPT or p15RS expression with four selected Wnt downstream genes in colon tissues using mRNA sequencing data from TCGA and GTEx databases. Pearson correlation coefficients (Pearson’s r) for each pair are labeled in the heat map. (**b**,**c**) RT-PCR verified the transcriptional level of selected Wnt downstream genes in SW480 cells with CREPT or p15RS KO (**b**) or overexpression (**c**). (**d**,**e**) Western blotting showed protein expression level of cyclin D1 and c-Myc in SW480 (**d**) and MGC803 (**e**) cell lines under normal condition or after starvation-stimulation treatment. Fold: relative protein expression normalized to the loading control and calibrated to the WT group under normal conditions. (**f**) Overexpression of CREPT promoted while p15RS inhibited Wnt signaling, especially under exogenous Wnt1 stimulation. (**g**) Overexpression of CREPT promoted while p15RS inhibited Wnt signaling, particularly under Wnt3a conditioned medium treatment. (**h**,**i**) CREPT promoted, but p15RS inhibited Wnt signaling in CREPT KO (**h**) or p15RS KO (**i**) HEK-293T cell lines. *** *p* < 0.001, ** *p* < 0.01, * *p* < 0.05, n.s., no significance.

Our previous studies have demonstrated that both CREPT and p15RS are involved in the regulation of Wnt/β-catenin signaling [23,29]. However, these studies demonstrated opposite roles of CREPT and p15RS in Wnt activated genes expression. In particular, Wnt signaling was accelerated by CREPT, but repressed by p15RS during tumorigenesis. To confirm whether Wnt activated genes are oppositely regulated by CREPT and p15RS, we performed a correlation analysis between the expression levels of *CREPT/p15RS* and those of Wnt signaling downstream genes in colon tissues obtained from TCGA and the GTEx database. Among the 565 samples analyzed, 141 samples (the top 25%) with high CREPT expression also exhibited high levels of Wnt target genes, including *CCND1*, *Myc*, *TCF7*, and *Axin2* [44]. However, in the 140 samples with low CREPT expression (the bottom 25%), these Wnt target genes were mostly expressed at low levels (Figure 4a, top panel). Conversely, samples with high p15RS expression exhibited low levels of Wnt target genes, while those with low p15RS expression generally showed high expression of these genes (Figure 4a, bottom panel). Pearson correlation analysis further revealed a positive correlation between CREPT expression and Wnt target gene expression and a negative correlation between p15RS expression and Wnt target gene expression (see r in Figure 4a). These results indicate that CREPT and p15RS oppositely regulate Wnt target gene expression.

Next, we performed RT-PCR experiments to verify the results from the public database. Upon stimulation with complete medium to activate intracellular Wnt signaling, the mRNA level of *CCND1*, *Myc*, *TCF7*, and *Axin2* decreased in CREPT KO cells, but increased in p15RS KO cells (Figure 4b). Consistently, the expression of these genes was upregulated in CREPT-overexpressing cells, but downregulated in p15RS-overexpressing cells (Figure 4c). Collectively, these results demonstrated the differential roles of CREPT and p15RS in the regulation of Wnt signaling.

Subsequently, we detected the expression of Wnt target genes at the protein level. To further characterize the roles of CREPT and p15RS in Wnt signaling, we examined the expression of downstream proteins under both basal and serum starvation-stimulation conditions. Western blot analyses showed that cyclin D1 was dramatically increased when p15RS was deleted, but maintained at low levels when p15RS was overexpressed under both basal and Wnt activation conditions in both SW480 (Figure 4d) and MGC803 (Figure 4e) cells. On the other hand, cyclin D1 was decreased when CREPT was deleted, but increased when CREPT was overexpressed in both cell lines (Figure 4d,e). Consistently, we observed that c-Myc expression was upregulated by CREPT, but downregulated by p15RS in these cells under different conditions, although the fold change varied (Figure 4d,e). Taken together, these results confirmed that CREPT is positively correlated and p15RS is negatively correlated with the expression of Wnt target genes at the protein level.

Finally, we assessed the transcriptional activity by canonical Wnt signaling using the SuperTop-Flash dual-luciferase reporter system in 293T cells. The results showed that overexpression of CREPT enhanced, but overexpression of p15RS impaired luciferase activity upon Wnt1 (Figure 4f) and Wnt3a (Figure 4g) stimulation. To avoid interference from endogenous CREPT and p15RS, we ectopically expressed either CREPT or p15RS in HEK-293T cells with CREPT or p15RS knockout. Similar results were observed under Wnt1 stimulation (Figure 4h,i). All these luciferase experiments indicated that CREPT enhanced, but p15RS inhibited the transcriptional activity of the Wnt/β-catenin pathway.

### 3.5. The CID Domain Is Crucial to the Function of CREPT and p15RS

**Figure 5:**
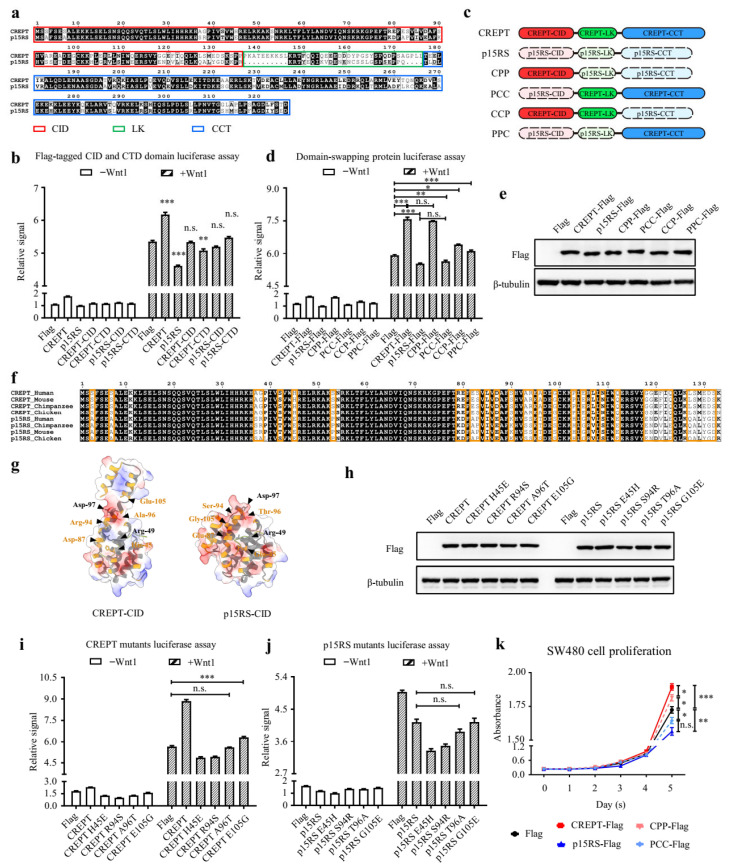
CID domains are responsible for the function of CREPT and p15RS. (**a**) Sequence alignment and domain architecture of human CREPT and p15RS. (**b**) Overexpressing the CID or CTD domain alone failed to promote or inhibit Wnt signaling. The significance of each group was assessed by pairwise comparison against the Flag vector control group. (**c**) Domain architecture schematic for CREPT, p15RS and domain-swapping recombinant proteins. (**d**) Dual luciferase assay showed the transcription activity of Wnt signaling when CREPT, p15RS, and the domain-swapping proteins were overexpressed. (**e**) Protein expression status of (**d**). (**f**) Sequence alignment of the CID domains of CREPT and p15RS across selected species. (**g**) Structural comparison of CREPT and p15RS CID domains. Key differential residues are labeled with their amino acid names and sequence positions. (**h**) Expression verification of CREPT, p15RS, and the site-specific mutant proteins. (**i**,**j**) Dual luciferase assay indicated the transcriptional activity of Wnt signaling when CREPT (**i**) or p15RS (**j**) related mutants were overexpressed. (**k**) Proliferation rates of SW480 cells after transient transfection with the indicated plasmids. *** *p* < 0.001, ** *p* < 0.01, * *p* < 0.05, n.s., no significance.

To decipher how CREPT and p15RS oppositely regulate transcription, we analyzed the sequence differences between these two proteins. Amino acid sequence alignment showed that both the CID domain (residues 1–136 at the N-terminus) and the CCT domain (residues 177–326 for CREPT and 163–312 for p15RS at the C-terminus) shared high sequence identity. However, the linker domain (residues 137–176 for CREPT and 137–162 for p15RS) varied dramatically, with two deletions of eight and four amino acids, respectively (Figure 5a). We speculated that the differences in their domain architectures might contribute to their opposing functions.

To verify this hypothesis, we expressed the isolated CID domains and CTD domains (linker plus CCT domains) and examined their effects on transcription using the dual-luciferase reporter system in response to Wnt signaling. The results showed that when expressed individually, the CID and CTD domains of CREPT failed to enhance transcription, and the corresponding domains of p15RS exhibited no transcriptional inhibitory effect either (Figure 5b). This result indicated that the effects of CREPT or p15RS on transcription depend on the structural integrity of the full-length proteins.

We therefore adopted a domain-swapping strategy to dissect the functional contribution of each individual domain. Specifically, we constructed four domain-swapping proteins, CPP (CREPT-CID+p15RS-linker+p15RS-CCT), PCC (p15RS-CID+CREPT-linker+CREPT-CCT), CCP (CREPT-CID+CREPT-linker+p15RS-CCT), and PPC (p15RS-CID+p15RS-linker+CREPT-CCT), by exchanging the corresponding domains between CREPT and p15RS (Figure 5c), and confirmed the correct expression of all Flag-tagged constructs (Figure 5e). Dual luciferase reporter assays showed that the CPP protein exhibited a promoting effect on luciferase activity, similar to full-length CREPT, while the PCC protein inhibited the transcriptional activity, similar to full-length p15RS (Figure 5d, CREPT-Flag vs. CPP-Flag; p15RS vs. PCC-Flag). These results suggested that exchange of the CTD domains between CREPT and p15RS had no impact on their individual functions in promoting or inhibiting transcription. In other words, the result implied that the CID domains were essential for determining the transcriptional activity of CREPT and p15RS. In addition, we observed that the CCP protein displayed significantly reduced capacity to promote Wnt signaling transcriptional activity, while the PPC protein lost the ability to suppress Wnt signaling transcriptional activity (Figure 5d, CREPT-Flag vs. CCP-Flag; p15RS-Flag vs. PPC-Flag). These results suggested that substitution of the CREPT CCT domain with p15RS CCT domain disarmed the transcription promoting function of CREPT. Reciprocally, substitution of the p15RS CCT domain with the CREPT CCT domain diminisher the inhibitory role of p15RS. Altogether, our results highlighted the crucial role of the CID domain in determining the functions of CREPT and p15RS in Wnt signaling, although the linker or CCT domains may also participate in this process.

To search for detailed differences, we aligned the CID domains from CREPT and p15RS in several closely related species. Differential residues, labeled with orange boxes, were highly conserved among species from chicken to human (Figure 5f). Interestingly, we found that four of these differential residues contributed to structural differences (PDB: 4Q94, 4JXT) (Figure 5g). In particular, Thr-96 in p15RS replaces Ala-96 in CREPT, leading to conformation alteration; Glu-45 and Ser-94 in p15RS substitute His-45 and Arg-94 in CREPT, which reverses the surface charge property from negative in p15RS to positive in CREPT; and Gly-105 in p15RS replaces Glu-105 in CREPT, which may disrupt the estrogen receptor recognition motif LXXLL (Figure 5g). Based on this information, we generated site-mutated proteins by interchanging residues 45, 94, 96, and 105 in both CREPT and p15RS (Figure 5h). We then detected changes in transcriptional activity using the dual-luciferase experiment. The result showed that all CREPT mutants, particularly the H45E and R94S mutants, lost the transcriptional promotion ability (Figure 5i). Interestingly, we observed that the mutants of p15RS in these residues retained the inhibitory ability, with a strong inhibition role in E45H and S94R mutants (Figure 5j). These results imply that these four residues are critical for the promoting role of CREPT, but not critical for the inhibitory function of p15RS. We speculate that the inhibitory role of p15RS might be due to the other sequence differences in the linker or the CCT domain.

Finally, we evaluated the function of CID domain in cell proliferation. Following transient expression of CREPT, p15RS, CPP, and PPC proteins in SW480 cells, we observed that cell proliferation was promoted by CPP, but inhibited by PCC, similar to the phenotypes observed in cells with CREPT or p15RS overexpression (Figure 5k). In summary, our findings demonstrate that the CID domains determine the functions of CREPT and p15RS in both Wnt signaling and cell proliferation. The functional disparities between CREPT and p15RS can be partially explained by their distinctive conformation and charge distribution in the CID domains.

### 3.6. CREPT Exhibits a Higher Capacity for Oligomerization Compared to p15RS

Besides the CID domain, the CCT domain is another essential functional domain of CREPT and p15RS. Previous studies have demonstrated that the CCT domain of p15RS contains a leucine zipper-like motif, which facilitates homologous interaction between p15RS molecules [37]. The high sequence similarity observed between the CCT domains of CREPT and p15RS prompted us to investigate whether CREPT could generate self-interaction or mutual-interacts with p15RS.

**Figure 6:**
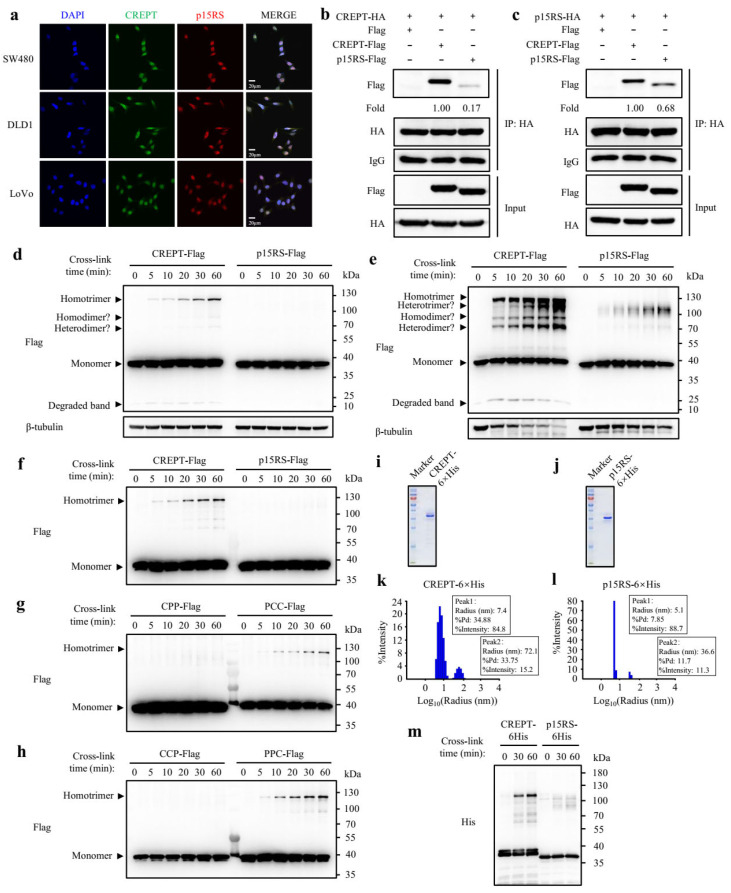
The CREPT CCT domain mediates its self-interaction and homo-oligomerization. (**a**) The co-localization of CREPT and p15RS in different colon cancer cell lines. (**b**) Immunoprecipitation analysis showed the interaction of CREPT-HA with CREPT-Flag or p15RS-Flag. (**c**) The interaction of p15RS-HA with CREPT-Flag and p15RS-Flag. Fold, IP/input ratio, with the highest ratio normalized to 1. (**d**,**e**) Cross-linking assay showed the oligomerization of CREPT and p15RS when treated with 0.1% (**d**) or 1% (**e**) formaldehyde for indicated time. (**f**–**h**) 0.1% formaldehyde cross-linking assay showed the oligomerization of indicated proteins. (**i**,**j**) Purified prokaryotic CREPT (**i**) and p15RS (**j**) proteins. (**k**,**l**) DLS assay demonstrated the oligomerization of CREPT and p15RS proteins in native status. (**m**) 0.1% formaldehyde cross-linking assay showed purified prokaryotic CREPT and p15RS proteins were able to form oligomers.

First, we performed immunofluorescent staining to detect the co-localization of endogenous CREPT and p15RS in multiple colon cancer cell lines, including SW480, DLD1, and LoVo. The results showed prominent co-localization of CREPT and p15RS in the nuclei of all tested cell lines (Figure 6a), indicating potential functional relevance and mutual interaction. Next, we ectopically co-expressed CREPT-HA with CREPT-Flag or p15RS-Flag in HEK-293T cells. Co-IP assays were then performed to identify proteins interacting with CREPT. Interestingly, although CREPT-HA interacted with CREPT-Flag and p15RS-Flag, the interaction strength of CREPT self-association (CREPT-HA and CREPT-Flag) was markedly stronger than its heterologous interaction with p15RS (CREPT-HA and p15RS-Flag) (Figure 6b). We further performed Co-IP assays to assess the interaction of p15RS-HA with CREPT-Flag or p15RS-Flag. The results revealed a moderate interaction between p15RS-HA and CREPT-Flag, while p15RS-HA exhibited markedly weaker self-interaction with p15RS-Flag (Figure 6c). Taken together, our results demonstrated that CREPT self-interaction was the strongest, followed by the heteromeric interaction between CREPT and p15RS, while p15RS self-interaction was the weakest among all detected combinations.

Based on the strong interaction observed between CREPT molecules, we speculated that CREPT might form oligomers. We ectopically expressed CREPT or p15RS in HEK-293T cells and performed a protein cross-linking experiment by treating the cell lysates using 0.1% formaldehyde for different durations. Western blot analysis revealed distinct bands between 100 kDa and 130 kDa for CREPT-Flag protein, corresponding to the trimeric size of the CREPT monomer. However, no high molecular weight bands were observed for p15RS-Flag even after 60 min of formaldehyde treatment (Figure 6d). When the formaldehyde concentration was increased to 1%, both CREPT and p15RS showed trimer bands. However, the high-molecular-weight bands of CREPT were significantly stronger than those of p15RS (Figure 6e). These data suggested that CREPT had a higher propensity to form oligomers than p15RS.

To further determine the specific domain responsible for protein oligomerization, we purified CREPT, p15RS, CPP, PCC, CCP, and PPC proteins expressed in HEK-293T cells using anti-Flag affinity beads and performed a cross-linking assay using 0.1% formaldehyde. Western blot results showed that CREPT, PCC and PPC exhibited homo-trimer bands, whereas p15RS, CPP and CCP showed no obvious oligomer bands (Figure 6f–h). Since CREPT, PCC and PPC all contain CREPT CCT domain, while p15RS, CPP and CCP all contain p15RS CCT domain in their C-terminal regions, we reasoned that the CCT domain is responsible for CREPT oligomerization.

To further exclude the possibility that other co-purified endogenous factors contribute to CREPT oligomerization, we purified human CREPT and p15RS from *E. coli* (Figure 6i,j). We measured the particle aggregation status of these proteins in native status via DLS assay. The results showed that the radius distribution of CREPT (%Pd = 34.88; Pd, polydispersity) was significantly broader than that of p15RS (%Pd = 5.1) (Figure 6k,l). This result indicated that CREPT existed in both monomeric and oligomeric forms, whereas p15RS predominantly existed as monomers in native conditions. Furthermore, cross-linking assays using 0.1% formaldehyde also showed that prokaryotically expressed CREPT formed trimers, while p15RS remained mostly monomeric (Figure 6m). This result confirmed that CREPT was oligomerized in native status relying on the CCT domain.

### 3.7. Oligomerization Is Indispensable for the Function of CREPT and p15RS

**Figure 7:**
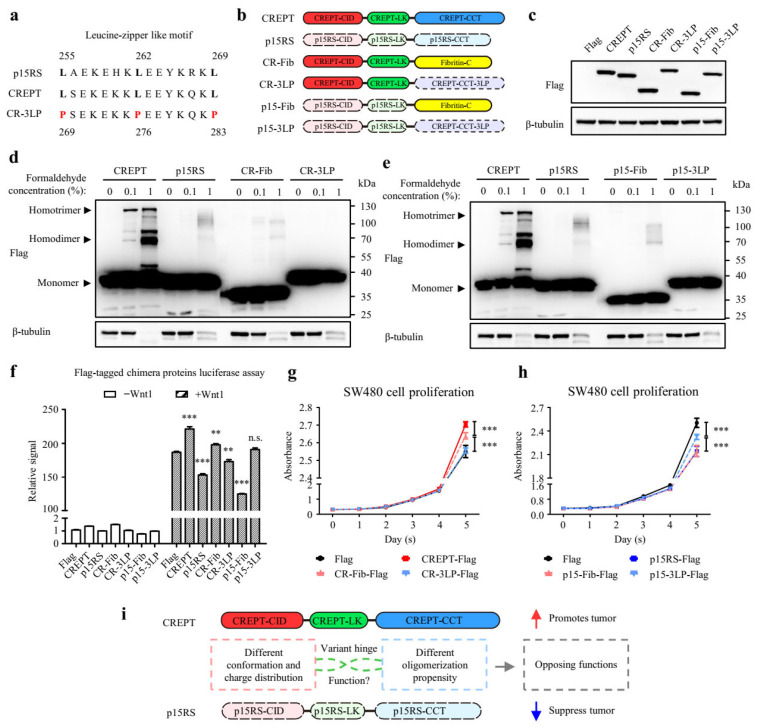
Oligomerization is required for the function of CREPT and p15RS. (**a**) Schematic diagram of 3LP mutation in the leucine zipper-like motif of CREPT and p15RS. (**b**) Domain structure illustration for CREPT, p15RS and chimeric proteins. (**c**) Protein expression status of CREPT, p15RS and chimeric proteins. (**d**,**e**) Cross-linking assay showed the oligomerization status of indicated proteins after a 30 min treatment with formaldehyde at concentrations of 0%, 0.1% and 1%. (**f**) The dual-luciferase reporter assay showed the transcriptional activity of Wnt signaling when indicated proteins were overexpressed. (**g**,**h**) Proliferation rates of SW480 cells after transient transfection with the indicated plasmids. (**i**) A proposed model demonstrating the functional and biochemical differences between CREPT and p15RS. *** *p* < 0.001, ** *p* < 0.01, n.s., no significance.

In general, protein oligomerization is a key regulator of their biological functions [45]. We speculated if oligomerization of CREPT and p15RS is crucial for their biological functions. To this end, we introduced the C-terminal region of T4 fibritin (referred to as Fib) to promote trimerization of specific proteins [46]. In addition, we generated L269P/L276P/L283P mutations of CREPT CCT domain (referred to as 3LP) (Figure 7a) to disrupt the normal protein oligomerization of CREPT. Accordingly, chimeric proteins, including CR-Fib (CREPT-CID+CREPT-linker+Fib), CR-3LP (CREPT-CID+CREPT-linker+3LP), p15-Fib (p15RS-CID+p15RS-linker+Fib), and p15-3LP (p15RS-CID+p15RS-linker+3LP), were used in our study (Figure 7b,c).

To validate our design, we assessed the oligomerization status of these chimeric proteins by formaldehyde cross-linking assay (Figure 7d,e). As expected, the CR-3LP and p15-3LP chimeric proteins, which contained three leucine to proline mutations, lost their oligomerization capacity. However, surprisingly, the CR-Fib and p15-Fib chimeric proteins, which contained a T4 fibritin C-terminal region, exhibited a similar level of trimerization to that of p15RS, which was significantly weaker than that of CREPT. These results further confirmed the strong propensity of CREPT to form oligomers.

Then, we conducted a dual-luciferase assay to measure the transcriptional activity of Wnt signaling in the presence of CREPT, p15RS, and the chimeric proteins. The results showed that the CR-3LP and p15-3LP chimeric protein, which lost oligomerization ability, lost the promotive or inhibitory effects on Wnt signaling as well. Additionally, the CR-Fib chimeric protein, which retained partial oligomerization ability, exhibited impaired promotion of Wnt signaling transcription (Figure 7f). These results revealed the importance of protein oligomerization in mediating the biological functions of CREPT and p15RS. We further examined the cell proliferation of SW480 cells upon expression of these chimeric proteins. The data indicated that CR-3LP chimeric protein completely lost the ability to enhance cell growth of wildtype CREPT, while the CR-Fib protein showed a diminished promotive effect (Figure 7g). Similarly, the inhibitory effect of p15RS on cell growth also relied on its oligomerization (Figure 7h). These results indicated that in addition to the CID domain, the CCT domain is also essential for the function of CREPT and p15RS.

Taken together, we conclude that both CID and CCT domains contribute to the biological functions of CREPT and p15RS, yet act through distinct mechanisms (Figure 7i). Despite high sequence similarity, the CID domains of CREPT and p15RS adopt distinct conformation and charge distribution. Additionally, the CCT domains facilitate protein oligomerization with different strengths. Differences in both CID and CCT domains collectively account for the functional divergence between CREPT and p15RS.

## 4. Discussion

In recent decades, multiple independent groups have reported the functions of either CREPT or p15RS in tumorigenesis [15,25,27]. Nevertheless, the detailed underlying mechanisms responsible for the divergent biological functions of these two paralogues remain unclear. In this study, we performed a systematic investigation to dissect the differences in biological functions and biochemical mechanisms between CREPT and p15RS. Initially, our results showed that CREPT and p15RS are evolutionarily closely related and both highly expressed in tumor tissues. However, CREPT and p15RS were associated with distinct prognostic outcomes in patients. Next, we validated that CREPT promoted, but p15RS inhibited cell proliferation and the Wnt signaling pathway. Subsequently, we adopted a domain-swapping approach to investigate the specific functions of the CID and CCT domains in CREPT and p15RS. Our results showed that both the CID domain and the CCT domain were crucial for the function of either CREPT or p15RS. In detail, the CID domain determines the promotive or inhibitory effects of CREPT or p15RS, while the CCT domain is responsible for protein oligomerization, which is another key factor in their functional implementation.

Phylogenetic tree analysis from this study and our previous work [25] identified CREPT and p15RS as paralogues derived from a common Rtt103-like ancestor. As paralogues, CREPT and p15RS exhibit numerous similarities, including conserved amino acid sequences and the biological processes in which they are involved [25]. Despite this, we observed pronounced differences in downstream gene expression between CREPT and p15RS when analyzing TCGA and GTEx sequencing data (Figure 4a and Supplementary Figure S1). These results enlighten us that proteins with similar primary sequences may also exhibit distinctive functions. Similar cases have been reported in AP-1 family in transcription regulation and R-spondin family in Wnt signaling [47,48]. In mechanistic studies of these proteins, specific domains that were responsible for protein–DNA interaction or protein–protein interaction were reported to be crucial for their biological functions [4,49].

In our study, we uncovered the dedicated mechanisms of the distinct biochemical properties of CREPT and p15RS. Their divergent biological functions can be attributed to differences in the CID and CCT domains, despite their high sequence conservation. For the CID domain, we identified several key residues for the function of CREPT and p15RS (Figure 5g,h). These residues, which differ between CREPT and p15RS, confer specific surface electric potentials and thereby drive their divergent biological behaviors. Although other residues may also contribute to the function of CREPT, these sites better explain the functional divergence of CREPT and p15RS.

Accumulating evidence has demonstrated that protein oligomerization is crucial for the regulation of multiple cellular processes [45,50], as well as for the function of CREPT [32]. Our results highlight that although CREPT and p15RS differ in oligomerization propensity, oligomerization is essential for their respective functions. We observed consistent outcomes when their CID domains were chimerically fused with the C-terminal of T4 fibritin, a heterologous domain unrelated to both proteins. In contrast, a three-amino-acid mutation that disrupts oligomerization severely impairs the functions of both proteins (Figure 7f–h). Primary sequence analysis showed that both CREPT and p15RS contain a leucine zipper-like motif within their CCT domains (Figure 7a). Structural prediction using DeepCoil software (https://toolkit.tuebingen.mpg.de/tools/deepcoil (accessed on 4 June 2026)) [51] also indicated that both proteins were capable of forming coiled-coil structures. However, our experimental evidence showed that CREPT exhibited a markedly stronger propensity to form oligomers than p15RS (Figure 6d,e). This observation is consistent with previous structural studies: despite extensive efforts, the CCT domain crystal structure has been determined for CREPT, yet remains unsolved for p15RS [32,52]. Recently, our group reported that CREPT mediates large-scale chromatin loop formation in triple-negative breast cancer (TNBC) [22]. We speculated that CREPT oligomerization, which largely relies on the CCT domain, also plays a vital role in mediating the genomic proximity. Previous structural studies reported the dimeric structure of CREPT CCT domain rather than a trimeric form [52]. Experimental and computational studies have shown that subtle changes in amino acid arrangement in the coiled-coil region can lead to pronounced topological differences [53]. We propose that the inconsistent oligomerization states reported across different studies may arise from differences in buffer conditions or the truncated protein sequences used. Differences in oligomerization capacity between CREPT and p15RS offer novel mechanistic insights into their divergent roles in transcriptional regulation and also offer implications for CREPT-targeted drug design.

Our study has several limitations. First, the short flexible linker regions of CREPT and p15RS were not fully investigated. We recently identified functionally important modifications in these regions, which will be studied in detail in our subsequent research. Second, we found that CREPT can modulate p15RS expression (Figure 3a,b and Figure 4b,c). This is not unexpected, since CREPT is known to regulate transcription, and p15RS may be among its targets [32,35]. Their coordinated expression suggests close evolutionary and functional linkage, which requires further exploration alongside our work on functional divergence.

## 5. Conclusions

This study provides a systematic parallel comparison of the functions and biochemical properties of CREPT and p15RS. Our results elucidate the distinct properties of their CID and CCT domains, providing mechanistic insights into why these two structurally similar proteins exert opposing functions during tumorigenesis. These findings offer novel implications for the development of CREPT-targeted antitumor drugs.

## Data Availability

The data supporting the findings of this study are available within the article.

## Supplementary Materials

The following supporting information can be downloaded at: https://www.mdpi.com/article/10.3390/cancers18121911/s1, Figure S1: Correlation analysis of *CREPT* or *p15RS* expression with Wnt downstream genes; Table S1: Primers used for RT-PCR.
