# Supplementary material for: Opposing Roles of CREPT and p15RS in Tumorigenesis via Differential Regulation of Wnt Signaling
Source: Cancers (Basel). 2026 Jun 11;18(12):1911. doi: 10.3390/cancers18121911 (PMC13297466; doi:10.3390/cancers18121911)
Supplement: Supplementary file 1 [file cancers-18-01911-s001.zip › supplemental matrials/Supplementary Figure S1.pdf]

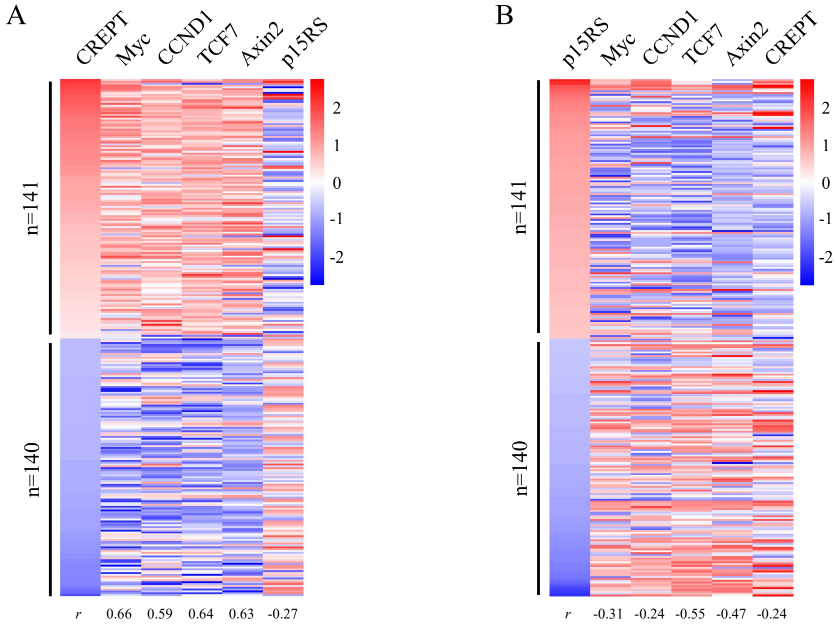

Figure S1. Correlation analysis of *CREPT* or *p15RS* expression with Wnt downstream genes. (A) Correlations between *CREPT* and four representative Wnt downstream genes (*Myc*, *CCND1*, *TCF7*, *Axin2*), as well as *p15RS*. (B) Correlations between *p15RS* and four representative Wnt downstream genes (*Myc*, *CCND1*, *TCF7*, *Axin2*), as well as *CREPT*. Data were obtained from the Xena platform using mRNA sequencing data of colon tissue from the TCGA and GTEx databases. Pearson correlation coefficients (Pearson's r) for each pair are labeled in the heat map.
